# Mefenamic Acid Poisoning Revisited: Central Nervous System Toxicity and Acute Kidney Injury

**DOI:** 10.7759/cureus.95848

**Published:** 2025-10-31

**Authors:** Fumiya Inoue, Yuji Okazaki, Toshihisa Ichiba, Akira Namera

**Affiliations:** 1 Department of Emergency Medicine, Hiroshima City Hiroshima Citizens Hospital, Hiroshima, JPN; 2 Graduate School of Public Health, St. Luke's International University, Tokyo, JPN; 3 Department of Forensic Medicine, Hiroshima University, Hiroshima, JPN

**Keywords:** acute kidney injury, drug concentration, mefenamic acid, seizure, self poisoning

## Abstract

Nonsteroidal anti-inflammatory drugs (NSAIDs) are a common cause of acute poisoning and often perceived as relatively benign in overdose. Mefenamic acid is unique among NSAIDs in that it is not available over-the-counter in many countries and may have a higher risk of central nervous system toxicity than other NSAIDs. Despite previous reports of seizures and renal dysfunction due to mefenamic acid poisoning, detailed information on the toxicity and the blood concentrations in recent case reports remains limited.

A 22-year-old woman presented with two episodes of generalized tonic-clonic seizures three hours after intentional ingestion of multiple analgesics, including 2.5 g (60 mg/kg) of mefenamic acid. On admission, she was agitated but neurologically intact. Initial laboratory findings showed normal renal function. She was admitted for observation. On day 3, she developed acute kidney injury (AKI) with a peak creatinine of 4.21 mg/dL. Her renal dysfunction gradually resolved without dialysis. Drug concentration analysis showed a serum mefenamic acid level of 42.2 µg/mL at admission (toxic threshold: 25 µg/mL). Based on clinical findings and drug concentrations, seizures and AKI were attributed to mefenamic acid poisoning.

Mefenamic acid poisoning can result in neurological and renal complications even at modest doses. Given its common use in dysmenorrhea and overlapping demographic with at-risk populations for overdose, clinicians should maintain a high index of suspicion and monitor closely when mefenamic acid ingestion is suspected. Further reports are needed to clarify relationships between serum concentrations, ingested dose, and clinical severity.

## Introduction

Nonsteroidal anti-inflammatory drugs (NSAIDs) are widely used analgesics and antipyretics available worldwide, including over-the-counter (OTC) preparations. Although NSAIDs, especially ibuprofen, account for a large proportion of acute poisonings, the absence of a specific antidote for acute NSAID poisoning and the generally favorable prognosis may lead clinicians to underestimate the clinical importance of NSAID poisoning [1].

Among the many NSAIDs in clinical use, mefenamic acid, commonly used for primary dysmenorrhea, has distinctive characteristics. Unlike other NSAIDs, it is not available OTC in many countries, including the United States, Europe, and Japan [2]. In addition, when compared to other NSAIDS, mefenamic acid poisoning has been associated with central nervous system (CNS) symptoms such as seizures [3]. It has been reported that approximately 11% of patients with mefenamic acid overdose develop seizures [4]. Although the exact mechanism underlying mefenamic acid-induced seizures remains unclear, it has been hypothesized that NSAIDs may lower the seizure threshold by inhibiting cerebral prostaglandin and/or thromboxane synthesis [5]. Despite this risk, detailed reports of severe mefenamic acid poisoning have been scarce since the 1990s, possibly because of the limited prescribing opportunities [6]. As such, the potential severity of acute poisoning may be underrecognized.

We report a case of polysubstance ingestion including mefenamic acid poisoning presenting with recurrent seizures and acute kidney injury (AKI), with serial measurements of serum drug concentrations.

## Case presentation

A 22-year-old woman, 144 cm in height and weighing 41 kg, presented to our emergency department (ED) in May 2024 after experiencing two episodes of generalized tonic-clonic convulsions, each lasting approximately one minute. She had no history of seizures, psychiatric disorders, or habitual medication use. Approximately three hours prior to ED arrival, she intentionally ingested the following medications prescribed for her mother: 10 tablets of 250 mg mefenamic acid (60 mg/kg), one tablet of 100 mg ibuprofen (2.5 mg/kg), 12 tablets of 60 mg of loxoprofen (18 mg/kg), and 14 tablets of 200 mg of acetaminophen (70 mg/kg). On arrival, her vital signs were as follows: Glasgow Coma Scale 13 (E4, V3, M6), body temperature 36.0°C, heart rate 110 beats per minute with sinus rhythm, blood pressure 124/60 mmHg, oxygen saturation 99% on room air, and respiratory rate 16 breaths per minute. Her GCS score remained at 13 for several minutes after the seizures stopped until she was transferred to the ED. She was in an agitated state but had no focal neurological deficits. Laboratory examinations showed normal creatine kinase and creatinine levels, no electrocyte abnormalities, and hyperlactatemia (Table 1).

**Table 1:** Laboratory examinations ED, emergency department; ALT, alanine transaminase; AST, aspartate transferase; LD, lactate dehydrogenase

	ED admission	Reference range
Venous pH	7.317	7.31 - 7.41
Venous pCO2	35.5	35 - 45 mmHg
Venous HCO3	18.2	22 - 46 mmol/L
Venous lactate acid	8.0	0.5 - 1.6 mmol/L
White blood counts	22400	3.3 - 8.6 x 10^3^/μL
Hemoglobin	13.4	13.7 - 16.8 g/dL
Platelet	46.4	15.8 - 34.8 x 10^4^/μL
AST	22	13 - 30 U/L
ALT	11	10 - 42 U/L
LD	275	124 - 222 U/L
Blood urea nitrogen	16	8 - 20 mg/dL
Creatinine	0.87	0.65 - 1.07 mg/dL
Creatinine kinase	182	59 - 248 U/L
C-reactive protein	0.012	< 0.14 mg/dL
Sodium	136.7	138 - 145 mmol/L
Potassium	3.1	3.6 - 4.8 mmol/L
Chloride	101.7	101 - 108 mmol/L
Magnesium	3.7	1.9 - 2.5 mg/dL
Phosphate	3.2	2.7 - 4.6 mg/dL
Glucose	157	73 - 107 mg/dL

Brain computed tomography showed no abnormal findings. We did not administer benzodiazepines because her agitated state gradually improved during her stay in the ED. Because the lactic acidosis was considered secondary to seizures, we provided supportive care. She was admitted with a diagnosis of suspected multi-drug overdose involving NSAIDs and acetaminophen. After admission to the intensive care unit, she gradually regained alertness over a few hours, and no further seizures were observed. We administered intravenous fluids and did not use any antiepileptic drugs. On day 1, she was able to resume oral intake; however, her creatinine level had increased to 1.48 mg/dL, while her magnesium level had returned to normal (10 hours after ED admission). Her magnesium level had normalized. On day 3, she developed pitting edema in both lower extremities, with her serum creatinine rising to 4.21 mg/dL. However, her kidney function gradually improved without hemodialysis, with her serum creatinine declining to 1.93 mg/dL on day 6. She was discharged without complications on day 6. At a follow-up visit on day 12, her kidney function completely recovered.

A high-performance liquid chromatograph/tandem mass spectrometer (1260 Infinity Liquid Chromatography System and 6420 Triple Quad Mass spectrometer, Agilent Technologies, Palo Alto, CA, USA) revealed that, among ingested drugs, only mefenamic acid levels at ED arrival exceeded toxic thresholds (Table 2) [7-9]. Based on the serum concentration and clinical presentation, we diagnosed mefenamic acid-induced recurrent seizures and AKI.

**Table 2:** Serum drug concentrations ED, emergency department * The ranges varied depending on the references. Serum sample for drug concentration analysis was obtained three hours after the overdose. Data on blood loxoprofen concentrations are limited. Drug concentrations of therapeutic, toxic, and lethal levels were referenced from references [7-9].

	Ibuprofen (µg/mL)	Loxoprofen (µg/mL)	Mefenamic acid (µg/mL)	Acetaminophen (µg/mL)
ED admission	26.7	22	42.2	38.4
10 hours after ED admission	10.4	8.5	0.34	10.1
Therapeutic levels	15-30	4-5	2-10 (20)^*^	10-25
Toxic levels	200	52-126	25	100-150
Lethal levels				200-300

## Discussion

The clinical course of this patient highlighted two important clinical issues: (i) mefenamic acid poisoning can initially present with CNS toxicity and may subsequently lead to AKI, and (ii) serious complications can occur even with a modest overdose of 2.5 g (60 mg/kg) of mefenamic acid.

Mefenamic acid poisoning may demonstrate distinctive toxicity characteristics that differ from those of other NSAID poisonings. While more than 20 different NSAIDs are currently available, mefenamic acid belongs to the fenamate class and demonstrates unique in vitro pharmacologic properties, including direct anti-enzymatic activity, which may contribute to its distinct toxicity profile [3]. Although ibuprofen overdose is the most common NSAID poisoning, its clinical course is typically mild, with most patients presenting with only gastrointestinal symptoms [1]. Severe toxicity, including renal or neurological impairment, generally occurs only with ingestion exceeding 400 mg/kg, and even in such cases, fatalities are exceedingly rare and often involve co-ingestions [1,10]. In contrast, although severe complications from mefenamic acid poisoning are also uncommon, the drug has a higher propensity to induce CNS symptoms [3]. Several reports from the 1980s documented CNS symptoms, including seizures, following mefenamic acid overdose [11]. In addition, only one previous case published in 1988 has documented both seizures and AKI with concurrent measurement of serum mefenamic acid concentrations [12]. Similarly, in our case, in addition to seizures early in the clinical course, our patient developed AKI with a peak serum creatinine level exceeding 4 mg/dL several days later. NSAID-induced AKI mainly results from prostaglandin inhibition, which removes the vasodilatory effect on renal arterioles and allows angiotensin II-mediated vasoconstriction [13]. Another strength of our case is the measurement of serum mefenamic acid concentrations. While the clinical course in our case is consistent with previous reports, further detailed reports are needed to better characterize mefenamic acid toxicity. This sequential development of neurological and renal complications in mefenamic acid poisoning, as seen in our case, is unusual but clinically important for guiding appropriate monitoring and observation.

Even a modest overdose of mefenamic acid may result in neurological or renal toxicity. For most NSAIDs, including ibuprofen, patients who ingest less than 100 mg/kg are generally considered to be at low risk of complications, and in asymptomatic cases, observation for approximately four hours is typically sufficient [1]. In contrast, while the toxicity profile of mefenamic acid appears to be dose-dependent, the exact thresholds for neurological or renal toxicity remain unclear. Previous literature suggests that seizures usually occur at doses exceeding 3.5 g or at serum concentrations above 100 µg/mL [3]. In addition, animal studies have demonstrated that seizures are induced at approximately 80 mg/kg [14]. Furthermore, there are few reports on the threshold for doses or serum concentrations that cause AKI in mefenamic acid poisoning. However, in our case, the patient ingested only 2.5 g (60 mg/kg), and the serum concentration at admission was 42.2 µg/mL. Despite these values being below the previously reported thresholds, seizures and AKI developed. In fact, it has been reported that the lowest dose to induce seizures is 2.5 g, and the lowest serum concentration is 21 µg/mL [3]. This suggests that individual susceptibility may play a larger role than previously assumed. In our case, although the co-ingestion of other substances may have contributed to the complications, the clinical course was most likely driven by mefenamic acid itself. The doses of ibuprofen and acetaminophen were within generally safe limits, and the serum concentrations of these drugs were not at toxic levels, suggesting that their impact was minimal. The toxic level of loxoprofen, however, remains poorly defined, making it difficult to assess its precise contribution. Given that the ingested loxoprofen was 720 mg, it is generally considered unlikely to have played a major role. Taken together, our findings support the need for caution, as even seemingly non-toxic doses of mefenamic acid may result in serious complications, including in the context of multi-drug ingestion.

## Conclusions

Mefenamic acid poisoning can result in neurological and renal complications even with a modest overdose, particularly when considering individual susceptibility and the context of multi-drug ingestion. Given the scarcity of recent detailed case reports, clinicians may underestimate the potential risks associated with mefenamic acid poisoning. Mefenamic acid is commonly used for dysmenorrhea, and this patient population (i.e., young women) overlaps with those at risk for overdose involving OTC preparations. In cases of overdose with analgesics and antipyretics, it is essential to obtain a detailed history of the specific drugs ingested, including the possibility of mefenamic acid, and to perform close monitoring for several days to detect the development of AKI. Because this is a single case report, the generalizability is limited. Therefore, further accumulation of case reports is warranted to better define the thresholds for neurological and renal toxicity.
